# Close of Volume First

**Published:** 1880-12

**Authors:** 


Close of Volume First.—The present issue ends the first volume of the *Independent Practitioner*, and it is but right and proper that we should say a few valedictory words to our readers and friends. This month (December) not only closes the volume of the Journal but records the last of an eventful year. In retrospecting the past twelve months there are probably very few of us who are entirely satisfied with what we have said, or the work we have accomplished; and there are none of us, perhaps, who do not feel that we could do better, and accomplished a greater amount of good for ourselves and others, could we but live the past over again !

We are conscious of many short comings in our work thus far, and it is our purpose to direct our efforts in such a way as to avoid them in the next year's duty to our readers and friends. To err is human, and probably no work has ever been accomplished that was entirely free from errors of omission, or commission. We promise our friends however, that every endeavor which we are capable of using, under the light that past experience affords will be directed towards rendering the *Independent Practitioner* one of the most useful and interesting Journals published in this country. We cannot close this brief editorial paragraph without returning our grateful thanks to all the friends and patrons of the Journal who have so generously extended to it the aid of their means and pens, and we feel we can safely invoke their continued assistance, in both these respects, for the new volume. A glance at the list of contributors of original communications to the first volume will show a most gratifying number of able writers, many of whom are justly regarded among the leading members of the profession. And in this connection, it affords us great pleasure to add, that we have been promised original articles for the next volume by a number of new contributors of the highest standing in the profession.

With best wishes for the happiness and prosperity of all our readers and friends for the incoming new year, who have so liberally extended their support, we bring our valedictory editorial to a close.

